# The Impact of Sodium Glucose Transporter 2 Inhibitors on Renal Parenchymal Oxygenation: An Unsettled Issue?

**DOI:** 10.34067/KID.0000001089

**Published:** 2026-03-26

**Authors:** Samuel N. Heyman, Zaid Abassi

**Affiliations:** 1 Department of Medicine, Hadassah Hebrew University Hospital, Jerusalem, Israel; 2 Ruth and Bruce Rappaport Faculty of Medicine, Technion-IIT, Haifa, Israel; 3 Department of Vascular Surgery, Rambam Health Care Campus, Haifa, Israel

**Keywords:** acute kidney failure, AKI, acute renal failure, hypoxia, kidney failure, renal ischemia, renal morphology, renal pathology, renal protection, SGLT2 inhibitors

Over the last decade, sodium glucose transporter 2 inhibitors (SGLT2i) revolutionized the management of type 2 diabetes, dramatically improving cardiovascular morbidity and mortality and markedly attenuating the progression of CKD and heart failure in diabetics as well as among nondiabetic patients.

Cardio-renal protection provided by SGLT2i is likely mediated by a combination of different mechanisms, including its effects on glycemic control, on total body sodium and water balance, and on various cellular metabolic pathways and cross-cellular signals mediating cell energetics, survival, inflammation, and fibrosis. By reducing proximal tubular reabsorption, SGLT2i decrease the metabolic activity at the renal cortex, required for sodium-glucose cotransport, responsible for some 30% of renal oxygen requirements. In addition, these agents promote a shift in systemic and renal fuel metabolism from carbohydrate utilization toward increased ketogenesis, which is considered a more energy-efficient substrate for renal tissues under stress conditions.^1^ This metabolic shift may further contribute to improved renal energy efficiency and oxygenation. Furthermore, reduced sodium/glucose cotransport at the renal cortex enhances sodium delivery to the macula densa, activating the tubuloglomerular feedback mechanism. Consequent reversal of glomerular hyperfiltration is also expected to diminish tubular transport load, hence improving renal oxygenation.

On the other hand, SGLT2i cause a shift of tubular transport activity from proximal to distal tubular segments. Proximal tubular transport inhibition enhanced downstream solute delivery and is expected to increase distal tubular transport and oxygen consumption, especially by thick ascending limbs.^2^ Indeed, acutely administered phlorizin (a nonselective SGLTi) restored reduced cortical pO_2_ in streptozotocin-induced diabetic rats and intensified medullary hypoxia both in diabetic and in control rats, with an overall decline in total renal oxygen consumption.^3^ Such intensified medullary hypoxia with SGLT2i might be clinically significant, likely explaining the rise in biomarkers of distal tubular injury in diabetic patients hospitalized with AKI while on SGLT2i, without biomarker evidence for proximal tubular damage.^4^

With that in mind, we have recommended caution in patients on SGLT2i, whenever there is a risk of intensified medullary hypoxic stress, such as the administration of radiocontrast agents or non steroidal anti-inflammatory drugs. However, unexpectedly, most studies indicate a reduced risk of AKI among patients chronically treated with SGLT2i. Can this indicate that the hypothesis of threatened medullary structures with SGLT2i by intensified regional hypoxia is wrong? Or could medullary hypoxia adaptation in patients chronically given gliflozins confers protection against acute hypoxic stress? Or alternatively, could there be a renal protective impact through improved cortical oxygenation or by mechanisms unrelated to oxygenation?

Another relevant parallel enigma is what causes erythrocytosis in patients given SGLT2i. While there is evidence that improved cortical oxygenation could recruit erythropoietin (EPO) producing cortical interstitial fibroblasts through hypoxia inducible factor-dependent tubulointerstitial cellular crosstalk, intensified hypoxia with SGL2i at the cortico-medullary junction can directly stabilize hypoxia inducible factor-2 signal in the canonical hypoxia-mediated pathway to generate EPO by EPO-producing interstitial cells at this region.^5^

Thus, revealing the effect of SGLT2i on renal cortical and medullary oxygenation in humans is essential for a better understanding of these unsettled issues. Blood oxygenation level dependent (BOLD) magnetic resonance imaging (MRI) is a noninvasive technique that enables the quantitation of parenchymal deoxygenated hemoglobin at specific target regions, illustrating changes in renal cortical and medullary pO_2_. In the current issue of *Kidney360*, Russo *et al*.^6^ applied this methodology to determine changes in renal oxygenation following chronic administration of SGLT2i. Eight diabetic and 12 nondiabetic patients with CKD (eGFR 25–75 ml/min per 1.73 m^2^) on maximally tolerated renin-angiotensin-aldosterone axis (RAAS) blockade were subjected to dapagliflozin or to placebo, with BOLD MRI performed at baseline and 12 weeks after the initiation of treatment. In this study, dapagliflozin did not affect cortical oxygenation, whereas medullary oxygenation increased, as compared with a placebo-treated group.^6^ An increase in medullary oxygenation was more pronounced in nondiabetic and younger patients, with lower systolic blood pressure and with smaller abdominal circumference, and was directly affected by the use of diuretics.

The effect of gliflozins on renal oxygenation, assessed by BOLD MRI, has been explored in few additional studies in humans, providing inconsistent outcomes (Table 1). Zanchi *et al*. studied the effect of acute and acute-on-chronic (1 month) administration of empagliflozin on renal oxygenation profile, 3 hours after drug administration in 30 healthy subjects with an eGFR of 113 ml/min per 1.73 m^2^.^7^ Cortical and medullary oxygenation determined by BOLD MRI were unaffected by acute or acute-on-chronic administration of empagliflozin and were comparable with control patients given placebo in a double-blinded fashion. Laursen *et al*. explored the acute effect of a very high dose of dapagliflozin versus placebo in 15 patients with insulin-dependent diabetes mellitus with albuminuria and a mean eGFR of 73 ml/min per 1.73 m^2^.^8^ Notably, 87% of the patients were treated by inhibitors of the RAAS, and 33% were managed by loop diuretics. In this study, dapagliflozin increased cortical oxygenation by 6 hours but had no effect on medullary pO_2_. Total and regional blood flows remained unaffected, underscoring the profound impact of oxygen expenditure on renal oxygenation profile. By contrast, Gullaksen *et al*. studying 20 patients with non-insulin-dependent diabetes mellitus chronically treated with empagliflozin for 32 weeks (70 and 50% of them managed by RAAS inhibitors and diuretics, respectively) found that while cortical oxygenation remained unchanged as compared with baseline measurements, medullary oxygenation significantly declined.^9^ Zhang *et al*., on the other hand, found that as compared with placebo, empagliflozin treatment for 6 weeks in 27 patients with early non-insulin-dependent diabetes mellitus increased medullary oxygenation without changes in cortical oxygenation,^10^ findings comparable with the currently data, presented by Russo *et al*.^6^

**Table 1:** Effect of SGLT-2 inhibitors or renal oxygenation in experimental and clinical studies

Study	Study Population	Regional Perfusion (ml/100 g per min)		Renal Oxygenation	
		Cortex	Medulla	Cortex	Medulla
O'Neill^3^	Streptozotocin-induced diabetic (*n*=9) and control rats (*n*=12) acutely treated with phlorizin			↑ in diabetic animals ↔ in nondiabatic rats	↓ in both diabetic and nondiabetic animals
Laursen^8^	15 individuals with IDDM and albuminuria: acute effect of high-dose dapagliflozin (50 mg) versus placebo	↔	↔	↑	↔
Gullaksen^9^	20 patients with NIDDM chronically treated with empagliflozin (10 mg) for 32 wk	↓	↔	↔	↓
Zhang^10^	27 patients with early NIDDM and 27 controls, chronically treated with empagliflozin (10 mg) versus placebo for 6 wk			↔	↑
Zanchi^7^	45 healthy subjects given emphagliflozin (10 mg): acute (3 h) and long-term chronic effect (30 d)			↔	↔
Russo^6^	Patients with CKD NIDDM and nondiabetics chronically treated with dapagliflozin (10 mg, *n*=11) or placebo (*n*=9) for 12 w			↔	↑

IDDM, insulin-dependent diabetes mellitus; NIDDM, non-insulin-dependent diabetes mellitus; SGLT-2, Sodium Glucose Transporter 2.

Thus, the evaluation of the effect of SGLT2i on renal oxygenation profile in humans frustratingly provides inconsistent findings. Technical diversities and factors, such as tissue composition, hematocrit, and changes in regional blood volume, may limit the usefulness of BOLD MRI studies. But to large extent, this may reflect the heterogeneity of studied patients. Whereas O'Neil experiments were conducted in a defined homogenous cohort of rodents, diverse outcomes in human studies possibly reflect different modes of drug administration (acute versus chronic, varied drugs, and doses) and patient heterogeneity (diabetics versus nondiabetic subjects, diabetes duration, management and control, the existence and degree of CKD, proteinuria and renal structural changes, the status of systemic hemodynamics and cardiovascular performance, sodium balance and hydration state, sympathetic activity, age, sex, and so forth), all of which may have profound effect on both renal hemodynamics and on oxygen expenditure along various nephron segments. Furthermore, coadministered medications, such as RAAS inhibitors, loop diuretics, and non steroidal anti-inflammatory drugs, also have profound effect on renal microcirculation and on regional oxygen expenditure. The supplementary figure in Russo's report illustrates the heterogenous response of renal MRI, with erratic changes in R2* within the study groups. This is not unexpected with the wide range of baseline kidney function and other clinical parameters of the evaluated individuals in this study.

To better assess noninvasively the effect of SGLT2i on renal oxygenation in humans, much larger and definitely more homogenous groups of patients are needed, controlled for the above-mentioned confounders. Without these data we are currently unable to relate renoprotective properties of SGLT2i to improved renal oxygenation or to the induction of hypoxia adaptive responses, to assess the risk of hypoxic AKI after its acute administration in specific clinical setups, or to define the mechanism by which gliflozins induce erythrocytosis.
